# Validation of the German revised version of the program in palliative care education and practice questionnaire (PCEP-GR)

**DOI:** 10.1186/s12904-017-0263-3

**Published:** 2017-12-28

**Authors:** Katharina Fetz, Ursula Wenzel-Meyburg, Christian Schulz-Quach

**Affiliations:** 1 Chair of Research Methodology and Statistics in Psychology, Department of Psychology & Psychotherapy, Faculty of Health, Witten/Herdecke University, Witten, Germany; 2 Interdisciplinary Centre for Palliative Medicine, Medical Faculty, Heinrich Heine University Dusseldorf, Dusseldorf, Germany; 3 Institute of Psychiatry, Psychology and Neuroscience, King’s College London, London, UK; 4 South London and Maudsley NHS Foundation Trust, London, UK; 5 Liaison Psychiatry, St. Christopher’s Hospice, London, UK

**Keywords:** Palliative care, Medical education, Evaluation, Teaching assessment, Psychometric evaluation, Validation, Principle component analysis, Reliability

## Abstract

**Background:**

The evaluation of the effectiveness of undergraduate palliative care education (UPCE) programs is an essential foundation to providing high-quality UPCE programs. Therefore, the implementation of valid evaluation tools is indispensable. Until today, there has been no general consensus regarding concrete outcome parameters and their accurate measurement. The *Program in Palliative Care Education and Practice Questionnaire* (German Revised Version; PCEP-GR) is a promising assessment tool for UPCE. The aim of the current study was to evaluate the psychometric properties of PCEP-GR and to demonstrate its feasibility for the evaluation of UPCE programs.

**Methods:**

The practical feasibility of the PCEP-GR and its acceptance in medical students were investigated in a pilot study with 24 undergraduate medical students at Heinrich Heine University Dusseldorf, Germany. Subsequently, the PCEP-GR was surveyed in a representative sample (*N* = 680) of medical students in order to investigate its psychometric properties. Factorial validity was investigated by means of principal component analysis (PCA). Reliability was examined by means of split-half-reliability analysis and analysis of internal consistency. After taking into consideration the PCA and distribution analysis results, an evaluation instruction for the PCEP-GR was developed.

**Results:**

The PCEP-GR proved to be feasible and well-accepted in medical students. PCA revealed a four-factorial solution indicating four PCEP-GR subscales: *preparation to provide palliative care,* *attitudes towards palliative care,* *self-estimation of competence in communication with dying patients and their relatives* and *self-estimation of knowledge and skills in palliative care.*

The PCEP-GR showed good split-half-reliability and acceptable to good internal consistency of subscales. *Attitudes towards palliative care* slightly missed the criterion of acceptable internal consistency. The evaluation instruction suggests a global PCEP-GR index and four subscales.

**Conclusions:**

The PCEP-GR has proven to be a feasible, economic, valid and reliable tool for the assessment of UPCE that comprises self-efficacy expectation and relevant attitudes towards palliative care.

## Background

Palliative care is a growing field in medical education in numerous European medical faculties. The number of structured palliative care programs in medical education curricula is increasing [1]. In Germany, palliative care was integrated into the Medical Licensure Act for Physicians (ÄAppO) in 2009 and palliative care education for undergraduate medical students was subsequently incorporated into medical education curricula [2, 3]. Since 2013, there has been a formal obligation to teach and assess undergraduate medical students in palliative care skills and knowledge. There is an international [4] and national [5] consensus regarding palliative care curricula including recommendations on the structures and processes of medical undergraduate education and the enhancement of its quality.

Nevertheless, there is still evidence for limited knowledge in undergraduate medical students in Germany concerning palliative care [6–9]. A recent study found that only 47% of faculties include bedside teaching and only 59% include real patient contact in their palliative care curricula [10]. The majority of curricula focus on cognitive teaching methods such as lectures (93%) and seminars (75%). Skills and knowledge concerning palliative care are mainly assessed via multiple-choice tests (84%), possibly due to a lack of personnel resources. Consequently, the major part of undergraduate medical education in palliative care is focused on teaching factual knowledge rather than on imparting affective and communication skills.

But there is also evidence that, besides cognitive aspects and factual knowledge concerning the treatment of dying patients, affective learning objectives and attitudes towards dying patients are of major importance with regard to palliative care skills [11, 12]. A positive attitude towards terminally ill and dying patients as well as a positive attitude towards death seem to be essential with regard to an adequate professional physician role model [6, 11, 13]. Unconscious ambivalent and negative emotions towards death and dying and avoidance strategies may cause a decrease in the quality of medical practice [14–16].

Consequently, to investigate the quality and effect of undergraduate palliative care education, there is a need for suitable and rigorous evaluation methods concerning palliative care education outcomes which also include affective and attitude-related aspects. So far, little attention has been paid to the evaluation of palliative care programs [17–19]. How to adequately assess affective learning objectives and relevant attitudes in undergraduate medical students is an issue that has only rarely been investigated empirically. Evaluation studies mainly focus on medical students’ attitudes towards teaching contents and didactic approaches, cognitive knowledge as well as subjective self-estimation in skills and competencies [20].

There is still no general consensus on UPCE evaluation outcome parameters and measurement tools [21]. UPCE evaluation studies often use self-administered, unvalidated and even unpublished assessment questionnaires resulting in heterogeneous outcome parameters and less than robust methodological quality of research. Even though there indeed exist evaluation tools for medical faculties [17, 22], they fail to provide any guidelines regarding the measurement of the effects of education programs in medical students. A recent systematic review [21] examined outcome measures in UPCE evaluation studies, with eleven studies conducted between 1990 and 2011 meeting the inclusion criteria (palliative care education evaluation, undergraduate medical students). Indicators for the effectiveness of UPCE programs were medical knowledge [23–26], attitudes related to palliative care [24, 26, 27] perception of confidence in issues related to palliative care [28, 29], frequency of experiences in providing palliative care [26] and attitudes and emotional reactions towards death and dying [24, 27, 30–33]. The authors conclude, that "no universally applicable validated questionnaire to assess the effectiveness of undergraduate palliative care education could be identified" and that "the increased focus by educational institutions on instilling palliative care skills in healthcare students necessitates the development of comprehensive and validated tools to evaluate the effectiveness of education initiatives" [21].

A promising evaluation tool is the *Program in Palliative Care Education and Practice questionnaire* (PCEP) [34]. It was originally developed for the assessment of a training program for palliative care skills at Harvard Medical School, USA. In addition to aspects of factual knowledge in palliative care treatment it also includes aspects of attitudes towards palliative care and perceived self-efficacy expectation [35] with regard to providing palliative treatment. Because of the lack of palliative care education evaluation tools in the German language, Schulz et al. [35] developed a short translated version of the PCEP questionnaire (PCEP German Revised; PCEP-GR) for the evaluation of an UPCE program at Witten/Herdecke university, Germany. They proposed four subscales in the PCEP-GR: *preparation to provide palliative care,* 1 *attitudes towards palliative care, self-estimation of competence in communication with dying patients and their relatives* and *self-estimation of knowledge and skills in palliative care.* To our knowledge, there exist, so far, no systematic analyses of factorial structure and psychometric properties of the PCEP-GR. In order to demonstrate its feasibility for the evaluation of undergraduate palliative care education and to contribute to the aim of the application of validated assessment tools in UPCE program evaluation, the current study aims to provide data concerning the application and practicality, as well as the factorial structure, validity and reliability of the PCEP-GR questionnaire.

## Methods

Ethical approval was obtained from the Ethics Committee of the Medical Faculty of Heinrich-Heine-University Dusseldorf, Germany (protocol no. 4876, date of approval 26.11.2014). The study was conducted in accordance with the Declaration of Helsinki on Ethical Principles for Medical Research Involving Human Subjects [36].

### PCEP

*The Harvard Medical School Program in Palliative Care Education and Practice* was developed in 2005 in order to address the need among physician and nurse educators for curricular development in palliative care [34]. In order to evaluate the success of the program, a questionnaire was developed by means of a multi-step process including expert panels and peer review. The development process was informed by adult learning theory [37] and the self-efficacy concept by Bandura [38]. It was developed for the measurement of pre and post competence in palliative care and consists of self-administered items measured on a 5-point Likert-scale (original items are reported in [34]; PCEP-GR items are depicted in Table 2).

In its original version items were focussing on both the self-perceived ability to provide palliative care and to teach palliative care-relevant issues. On the basis of the original item set, Schulz et al. [35] developed a short version of the PCEP questionnaire (PCEP-GR, 36 items) in the German language for the evaluation of a UPCE program for medical students at Witten/Herdecke university, Germany. Because of the fact that undergraduate medical students were the focus group of the questionnaire, only items focusing on the ability to provide palliative care were extracted. Items focusing on the ability to teach palliative care-relevant aspects were excluded from the item set.

The item selection was theory-based, proposing four subscales *preparation to provide palliative care*, *attitudes towards palliative care*, *self-estimation of competence in communication with dying patients and their relatives* and *self-estimation of knowledge and skills in palliative care*.

### Study samples

Prior to data collection using a representative sample of medical students for the purpose of validation of the PCEP-GR questionnaire, a pilot study was conducted in order to test aspects of feasibility of the PCEP-GR questionnaire and to gain baseline values. This was intended to establish whether the time and effort for applying the questionnaire would be manageable and to pre-test ease of integration into a larger undergraduate cohort. The pilot study sample included 24 medical students (19 female, 5 male) without prior knowledge in palliative care who participated in a mandatory elective blended-learning course with real-patient contact [39]. The mean age of participants was 24.79 (*SD* = 3.19; range = 20–32 years). The median semester of the surveyed students was 5 (range 1–11).

The representative sample of undergraduate medical students at Heinrich Heine University, Germany, includes three cohorts (*N* = 680) representing two complete intake years of undergraduate medical students at year five of their training. The first cohort (*n* = 337) completed the questionnaire in the summer semester of 2013, the second cohort (*n* = 222) completed it in the winter semester of 2013/2014 and the third cohort (*n* = 121) completed it in the summer semester of 2014. Students’ average age was 28 years. Participants’ average duration of studies was 11 semesters. Participants were 65.5% (*n* = 445) female and 34.5% (*n* = 234) male. Almost two thirds were female, thus corresponding to the gender distribution of all undergraduate medical students at the HHU Dusseldorf. Our age and gender distribution, as well as length of study furthermore corresponds with the general demographics of the population of medical students in Germany [40–42].

### Data collection

Data for the pilot study were collected pre and post of a mandatory elective blended-learning course with real-patient contact. The response rate of questionnaires was 100%. Data for the representative sample of medical students were collected after their participation in the elearning course *Palliative Care Basics* [43] and prior to a written examination on the topic of palliative care. The response rate of questionnaires was 97%.

### Evaluation of feasibility

In order to investigate PCEP-GRs feasibility characteristics, we referred to the British National Institute for Health Research Trials and Studies Coordinating Centre’s (NETSCC) definition of feasibility and pilot studies [44]. We collected descriptive information on the willingness of clinicians to recruit participants, the practicality of delivering the questionnaire in the proposed setting and the time needed to collect and analyse data (e.g. answering time, response rates, and staff needed to perform this evaluation), as well as the acceptability of the intervention to the users [45].

### Statistical analysis

All statistical analyses were performed using IBM SPSS 22 for Windows. Data were controlled for plausibility prior to descriptive and inferential analyses.

PCEP-GR sum scores were examined concerning normal distribution by means of Kolmogorov-Smirnov-test. Descriptive statistics pre and post of the blended-learning course in the pilot study sample are reported. Sensitivity to change due to intervention of the PCEP questionnaire was investigated by means of t-test for dependent samples with measurement time as independent variable (pre-course vs. post-course) and the PCEP-GR sum score as dependent variable. Cohorts of the representative sample were controlled for systematic differences concerning PCEP-GR sum scores by means of univariate analysis of variance with cohort as independent variable (cohort 1, cohort 2, cohort 3) and PCEP-GR sum scores as dependent variables.

Data were controlled for suitability prior to factorial analysis by means of Kaiser-Meyer-Olkin measure of sampling adequacy. The hypothesis of a four-factorial version was tested using confirmatory principal component analysis assuming four main components. Sum scores were calculated for the extracted components and reported descriptively.

Split-half-reliability was estimated using the odd-even method. Spearman-Brown coefficients are reported. In order to evaluate the subscale reliability of the PCEP-GR questionnaire, internal consistency (Cronbach’s alpha) was calculated for each of the subscales.

### Evaluation instruction

After taking into consideration the results of principal component analysis and reliability analyses, an evaluation instruction for further use of the PCEP-GR questionnaire was developed.

## Results

### Descriptives

#### Pilot study

The response rate in the pilot study sample was 100%. PCEP-GR sum scores were normally distributed (*D* (23) = 150, *p* = .17). The pilot study sample showed a mean PCEP-GR sum score of 106.29 (*SD* = 18.08, range = 80–139) prior to the elective blended-learning course. After the course the mean PCEP-GR sum score was 124.38 (*SD* = 11.5; range = 104–145). The PCEP-GR sum score difference between pre and post elective blended-learning course was significant (*t* (23) = −4.55; *p* < .00; Cohen’s *d* = 1.20).

#### Representative sample

Cohort 1 had a mean PCEP-GR sum score of 111.67 (*SD* = 23.56). Cohort 2 had a mean PCEP-GR sum score of 114.76 (*SD* = 16.70). Cohort 3 had a mean PCEP-GR sum score of 115.10 (*SD* = 24.99). There were no systematic differences between the cohorts regarding their mean PCEP-GR scores (*F* (2, 668) = 1.83; *p* = .16).

#### Evaluation of feasibility

Recruitment of participants to our study was uncomplicated given the fact that administration of PCEP-GR was integrated into the end of module evaluation of a blended-learning UPCE seminar popular amongst medical students and offering training in communication with seriously ill patients [20, 43]. Analysis of the answering time revealed a mean answering time of 8.01 min (range 3.17–19.25). PCEP-GR was integrated into standard end of module student evaluation of the existing UPCE curriculum. No additional staff for administration and analysis of the outcomes was needed.

### Inferential statistics

#### Factorial validity

The obtained data were suitable for factorial analysis with a Kaiser-Meyer-Olkin criterion of .81. Confirmatory principal component analysis revealed a four-factorial solution supporting the hypothesis of four PCEP-GR questionnaire subscales: *preparation to provide palliative care, attitudes towards palliative care, self-estimation of competence in communication with dying patients and their relatives, self-estimation of knowledge and skills in palliative care.*

**Fig. 1:**
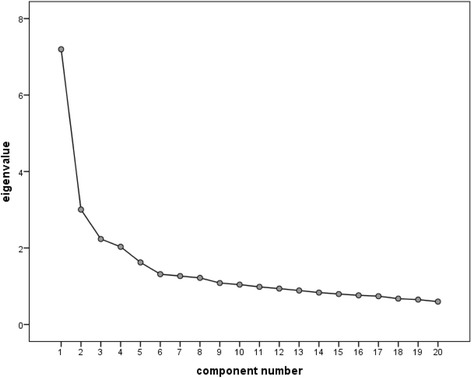
Screeplot of principal component analysis

For a scree plot of main components see Fig. 1. Eigenvalues and explained variance for each main component are presented in Table 1. The items of each main component and factor loadings are shown in Table 2. There were seven items which corresponded to other PCEP-GR subscales than proposed in the original version [35]. These items, as well as original and new subscales are presented in the additional files.

**Table 1:** Factor Strucuture

Main component^a^	Eigenvalue	Explained variance (%)
1. Preparation to provide palliative care	7.20	19.99
2. Attitudes towards palliative care	3.01	8.35
3. Self-estimation of competence in communication with dying patients and their relatives	2.23	6.21
4. Self-estimation of knowledge and skills in palliative care	2.03	5.64
total		40.20

^a^principal component analysis; varimax rotation, extraction criterion = 4 pc

**Table 2:** Factor structure of the new factor solution

Main component
1 Preparation to provide palliative care
1. Care for patients at the end of life.
2. Breaking bad news to a patient about his or her illness.
3. Managing patients’ emotional suffering at the end of life.
4. Discussing end-of-life care decisions, such as a DNR^b^ order, with a patient.
5. Managing ethical issues that arise in caring for patients near the end of life.
6. Helping family members during bereavement.
7. Discussing spiritual issues.
8. Discussing patient/ family psychosocial needs and concerns.
9. Addressing cultural differences related to end-of-life care.
10. Addressing age-related developmental differences in end-of-life care.
11. Responding to a patient’s question “Will I suffer much or have pain?”.
12. Your ability to determine patients’ needs.
13. Physicians have an obligation to tell patients when death is imminent.
2 Attitudes towards palliative care
1. There is little that can be done to ease the suffering of grief.^c^
2. The physician/nurse has a responsibility to provide bereavement care to the patient’s family after death.
3. It is not possible to tell patients the truth about a terminal prognosis and maintain hope.^c^
4. Psychological suffering can be as severe as physical suffering.
5. At their request, patients with terminal illnesses should be given whatever medications are necessary to relieve pain, even if the medications hasten death.
6. Talking about death tends to make patients with terminal illnesses more discouraged.^c^
7. Depression is not treatable in patients with terminal illnesses.^c^
8. The physician’s/nurse’s responsibility is to the patient; other professionals should deal with the needs of the family.^c^
9. Physicians/nurses have a responsibility to help patients prepare for death.
10. An interdisciplinary team approach to terminal illness treats patients’ medical needs better than conventional care.
3 Self-estimation of competence in communication with dying patients and their relatives
1. Depression is normal in patients with terminal illness.
2. Family members tend to interfere in the care of patients with terminal illnesses.^c^
3. Discussing possible symptoms of an incurable illness with patients.
4. Discussing possible symptoms of an incurable illness with the family.
5. Discussing death with patients.
6. Discussing the nearby death of patients with the family.
7. Discussing with the family after patients’ death.
8. Responding to a patient’s question “How long do I still have to live?”.
4 Self-estimation of knowledge and skills in palliative care
1. Managing pain in terminal illness.
2. Managing dyspnea or respiratory distress in terminal illness.
3. Your knowledge concerning the aetiology of palliative patients’ frequent symptoms.
4. Your ability to manage palliative patients’ frequent symptoms.
5. Your knowledge concerning the therapeutic and adverse effects of analgetics.

^b^DNR =do not resuscitate

^c^reversed item

#### Reliability

Analysis of split-half-reliability using the odd-even method revealed a Spearman-Brown coefficient of .90 (equal length). Analysis of internal consistency using Cronbach’s alpha was performed for each PCEP-GR subscale revealed by principal component analysis. Reliability coefficients for the PCEP-GR subscales are shown in Table 3.

**Table 3:** Reliability coefficients

Scale	Items	Internal consistency^d^
1. Preparation to provide palliative care	13	.83
2. Attitudes towards palliative care	11	.66
3. Self-estimation of competence in communication with dying patients and their relatives	7	.82
4. Self-estimation of knowledge and skills in palliative care	5	.75

^d^Cronbach’s alpha

#### Evaluation instruction

After taking into consideration the results of principal component analysis we suggest evaluating students’ perceived self-efficacy expectation in palliative care using four PCEP-GR subscales (*preparation to provide palliative care, attitudes towards palliative care, self-estimation of competence in communication with dying patients and their relatives,* and *self-estimation of knowledge and skills in palliative care).*

Each subscale is measured on a 5-point Likert-scale. The range for each subscale mean score is zero to five points, wherein higher scores indicate a higher parameter value for the respective subscale.

**Fig. 2:**
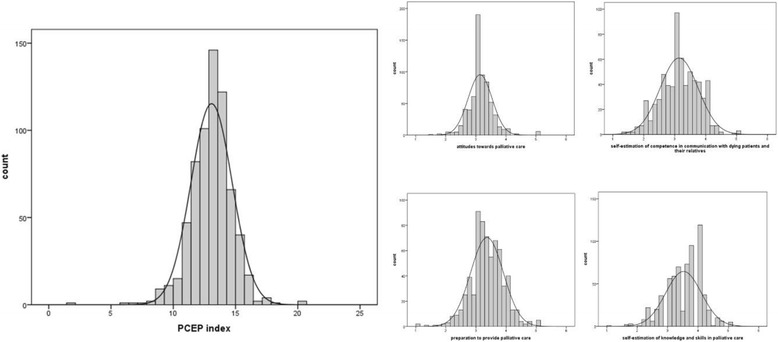
Distribution of PCEP-GR global index and subscales

As a global outcome parameter of UPCE programs, we furthermore suggest a *global PCEP-GR index* (sum score of mean subscales; range 0–20). The global PCEP-GR index is the sum score of the four PCEP-GR subscales (the sum of the mean subscales). Higher scores in the global PCEP-GR index indicate a higher degree of self-perceived competence in palliative care provision. The advantage of this index, in contrast to the PCEP-GR sum score lies in the fact that all of the four aspects measured by the PCEP-GR questionnaire are weighted equally. Mean values and standard deviation for the global PCEP-GR index and the PCEP-GR subscales are presented in Fig. 2.

In order to classify students’ global and subscale values, four ordinal classification groups were developed by means of descriptive analysis of mean, standard deviation and normal distribution. PCEP-GR subscale and global scores for the cohorts of the representative sample are shown in Table 4. Classification values for the PCEP-GR global index and sub scores are shown in Table 5.

**Table 4:** Mean PCEP-GR subscale scores and PCEP-GR index scores in the representative sample

Scale	Mean (SD)	Range	n
1. Preparation to provide palliative care	3.37 (0.54)	1.00–5.00	671
2. Attitudes towards palliative care	3.14 (0.40)	1.50–5.00	668
3. Self-estimation of competence in communication with dying patients and their relatives	3.14 (0.62)	1.38–5.00	658
4. Self-estimation of knowledge and skills in palliative care	3.53 (0.59)	1.00–5.00	667
PCEP-GR index	13.08 (1.66)	2.00–20.00	661

**Table 5:** Classification scores for PCEP-GR global index and subscales

Scale	Score	Class
PCEP-GR index^e^	< 10	Poor
	10–13	Acceptable
	13–16	Good
	> 16	Excellent
Subscales^f^	<1	Poor
	1–3	Acceptable
	3–4	Good
	> 4	Excellent

^e^Range 0–20

^f^Range 0–5

## Discussion

The evaluation of the effectiveness of UPCE programs is an essential foundation to providing high-quality UPCE programs, which is why the implementation of valid and robust evaluation tools is indispensable. The evaluation of UPCE programs should incorporate the broad range of knowledge, skills and attitudes that need to merge for medical students to achieve competence in the discipline of palliative care [17]. There is still no general consensus regarding concrete outcome parameters and their accurate measurement. The PCEP-GR questionnaire is a promising assessment questionnaire, which is why the aim of the current study was to demonstrate its feasibility for the evaluation of UPCE programs and to provide data concerning its factorial structure, validity and reliability.

Feasibility of PCEP-GR was assessed in a pilot study prior to the investigation of its psychometric properties. Considering its short answering time, high response rates and no additional staff involvement for administering PCEP-GR, we conclude this measure to be feasible for the evaluation of UPCE in large student cohorts.

PCEP-GR sum scores were normally distributed in the pilot study sample, suggesting that the PCEP-GR questionnaire covers a broad range of parameter values. There was a significant difference in mean PCEP-GR sum scores pre and post the blended-learning course, indicating a good sensitivity to change.

The factorial validity of the PCEP-GR was investigated by means of confirmatory principal component analysis. The hypothesis of a four-factorial solution including the subscales * preparation to provide palliative care, attitudes towards palliative care, self-estimation of competence in communication with dying patients and their relatives, and self-estimation of knowledge and skills in palliative care was therefore supported*. The analysis of the results of principal component analyses showed that there were several items corresponding to another subscale than proposed by the original authors. Interestingly, the items *physicians have an obligation to tell patients when death is imminent, depression is normal in patients with terminal illness* and *family members tend to interfere in the care of patients with terminal illnesses,* which were proposed to correspond to the subscale of *attitudes toward palliative care,* corresponded to the scales *preparation to provide palliative care* and *self-estimation of competence in communication with dying patients and their relatives* in the new factor-validated version. These results imply that attitude-related aspects are part of the feeling of preparation and self-estimation of competence. In summary, our results suggest a good factorial validity of the PCEP-GR questionnaire.

Analysis of split-half-reliability showed a good global reliability with a Spearman-Brown coefficient of .90. PCEP-GR subscales showed acceptable to good reliability indices in the analysis of internal consistency. *Attitudes towards palliative care* slightly missed the criterion of acceptable reliability. This may be due to the heterogeneous construct of attitudes towards palliative care. Overall, the PCEP-GR questionnaire can be considered to be a robust and reliable measurement tool.

In the evaluation instruction, we suggested four subscales and a global PCEP-GR index. For the subscales, a mean of raw values of the corresponding items was suggested. For the overall evaluation of UPCE programs, a global index (sum of subscales) was suggested. This procedure has the decisive advantage that all of the PCEP-GR aspects are weighted equally. On the other hand, it also depends on the assumption that all of these aspects are equally important regarding a global outcome parameter of UPCE. Since there is no general consensus on outcome parameters for UPCE, the question of hierarchical importance of different aspects of palliative care competence, skills and knowledge remains unsolved. There is, therefore, still a necessity for more research regarding this objective. Until further research may yield contrary findings, the PCEP-GR questionnaire seems to be a recommendable solution, considering its feasibility and psychometrical properties.

### Limitations

Despite the advantages of the PCEP-GR questionnaire, it must be conceded that questionnaire-based evaluation of UPCE in medical students has its limitations. In addition to attitudes towards palliative care and self-efficacy expectation, the construct of competence in palliative care includes also practical aspects with regard to providing palliative care and communicating with patients and relatives. Consequently, it would be advisable to supplement a questionnaire-based evaluation of UPCE programs by means of *Objective Structured Clinical Examinations* (OSCE) [46, 47] in order to gain valuable and more robust behavioural outcome data [28, 48, 49] Due to limited resources, however, this option is rarely feasible at university medical centres. Against the background of these circumstances and restrictions, questionnaire-based evaluation tools incorporating aspects of attitudes and self-efficacy expectation constitute a good interim solution for the evaluation of UPCE programs. It has furthermore to be mentioned that, due to unavailable sample characteristics, no further analyses of systematic differences in PCEP-GR scores concerning age and gender were possible. In the current study we were not able to assess PCEP-GR’s test-retest reliability since there was only a single time point of measurement. It was also not possible for us to investigate PCEP’s known-group validity, discriminant and convergent validity. Furthermore, our pilot study did not specifically focus on economic benefits although we were able to show that the application and administration of PCEP-GR can be easily integrated into large student cohorts without additional staffing support. There is more research needed to develop PCEP-GR, assess its psychometric properties further and to specifically evaluate its economic benefit.

## Conclusions

The PCEP-GR questionnaire has proven to be a feasible, economic, valid and reliable tool for the assessment of UPCE in medical students that also comprises the aspects of self-efficacy expectation and relevant attitudes towards palliative care.

## Abbreviations

ÄAppO: Medical licensure act for physicians
M: Mean
NETSCC: National Institute for Health Research Trials and Studies Coordinating Centre’s
OSCE: Objective structured clinical examination
PC: Palliative care
PCA: Principal component analysis
PCEP: Palliative care education and practice questionnaire
PCEP-GR: Palliative care education and practice questionnaire German revised version
SD: Standard deviation
UPCE: Undergraduate palliative care education

## Footnotes

1 [37] used the term *willingness to provide palliative care* for this subscale due to back translation from the German version. It was changed to *preparation to provide palliative care* after consultation with the language editor.
